# Menin Inhibitors in Acute Myeloid Leukemia: From Molecular Rationale to Clinical Practice

**DOI:** 10.3390/jpm16100534

**Published:** 2026-10-19

**Authors:** Caterina Alati, Matteo Molica, Martina Pitea, Gaetana Porto, Violetta Marafioti, Laura Giordano, Laura De Fazio, Marco Rossi, Massimo Martino

**Affiliations:** 1 Hematology and Stem Cell Transplantation, Cellular Therapies Unit (CTMO), Department of Hemato-Oncology and Radiotherapy, Grande Ospedale Metropolitano Bianchi-Melacrino-Morelli, 89133 Reggio Calabria, Italy; caterina.alati@ospedalerc.it (C.A.); porto.tania25@gmail.com (G.P.); violetta.marafioti@ospedalerc.it (V.M.); giordanolau@gmail.com (L.G.); dr.massimomartino@gmail.com (M.M.); 2 Department of Hematology-Oncology, Azienda Universitaria Ospedaliera Renato Dulbecco, 88100 Catanzaro, Italy; molica@bce.uniroma1.it (M.M.); defalaura@gmail.com (L.D.F.); rossim@unicz.it (M.R.)

**Keywords:** menin inhibitors, AML, KMT2A rearrangement, NPM1 mutation, revumenib, ziftomenib, bleximenib, enzomenib, targeted therapy, differentiation syndrome

## Abstract

**Background**: Acute Myeloid Leukemia (AML) harboring KMT2A rearrangements (KMT2Ar) or NPM1 mutations (mNPM1) constitutes a substantial and molecularly coherent subset of AML characterized by aberrant activation of homeobox (HOX) gene programs driven by the menin–MLL transcriptional complex. Disruption of this interaction through small-molecule menin inhibitors represents a compelling therapeutic strategy. **Methods**: We conducted a narrative (non-systematic) review of the clinical literature on four menin inhibitors—revumenib, ziftomenib, bleximenib, and enzomenib—as monotherapy and in combination regimens, identified through PubMed/MEDLINE and conference abstract searches (ASH, EHA) without a pre-specified systematic search protocol, as detailed in the Introduction. **Results**: Revumenib received FDA approval in November 2024 for relapsed/refractory (R/R) acute leukemia with a KMT2A translocation (an indication not restricted by lineage; the pivotal cohort included AML, ALL and MPAL), demonstrating a CR + CRh rate of 21.2% in the pivotal AUGMENT-101 trial, and a second FDA approval followed in October 2025 for R/R mNPM1 AML. In R/R mNPM1 AML (cohort 2C), an overall response rate (ORR) of 46.9% was observed. Ziftomenib achieved its primary phase II endpoint in R/R mNPM1 AML (CR/CRh 23%, *p* = 0.0058 vs. historical control) and received FDA approval (as Komzifti) in November 2025 for the same indication. Combination strategies with venetoclax/azacitidine have shown high response rates in small, non-randomized newly diagnosed cohorts (ORR approximately 88–94%), although the contribution of the menin inhibitor cannot be established without randomized data. Bleximenib and enzomenib show similarly encouraging early-phase signals. Resistance via acquired MEN1 mutations (M327I/V, G331R/D, T349M) has been identified in approximately 38.7% of revumenib-treated patients who progress after two or more cycles. Differentiation syndrome remains the most characteristic class-specific toxicity, occurring in approximately 11–28% of patients across agents (rates vary by cohort, data cut and prophylaxis strategy). **Conclusions**: Menin inhibitors represent a clinically important addition to the treatment of KMT2A- and mNPM1-driven AML, following the precedent set by FLT3 and IDH inhibitors. Combination approaches with hypomethylating agents and BCL-2 inhibition show particular promise, although no randomized controlled trial has yet demonstrated a survival benefit and current evidence derives predominantly from single-arm, early-phase studies. Ongoing randomized trials will define the definitive role of these agents across disease settings.

## 1. Introduction

### Overview of Acute Myeloid Leukemia

Acute myeloid leukemia (AML) is a clonal disorder of hematopoietic progenitor cells characterized by impaired differentiation and uncontrolled proliferation of myeloid blasts, with an annual incidence of approximately 4–5 cases per 100,000 population and a median age at diagnosis of 68 years [1]. The 2022 World Health Organization and International Consensus Classification schemes stratify AML by recurrent genetic abnormalities (e.g., PML::RARA, RUNX1::RUNX1T1, CBFB::MYH11, KMT2A rearrangements, NPM1 mutations, biallelic CEBPA mutations, TP53 alterations) and by defining cytogenetic/molecular lesions such as myelodysplasia-related gene mutations, reflecting substantial biological heterogeneity that underlies markedly different prognoses and treatment approaches [1,2]. The European LeukemiaNet (ELN) 2022 risk classification integrates cytogenetics and molecular findings (including FLT3-ITD allelic ratio, TP53, ASXL1, and RUNX1 status) into favorable, intermediate, and adverse risk groups, which guide decisions regarding consolidation chemotherapy versus allogeneic hematopoietic stem cell transplantation (HSCT) [2]. Standard frontline therapy for fit patients remains intensive induction chemotherapy (typically an anthracycline plus cytarabine, ‘7 + 3’), often followed by consolidation and, for higher-risk disease, allogeneic HSCT; for older or unfit patients, hypomethylating agents (azacitidine, decitabine) combined with the BCL-2 inhibitor venetoclax have become the standard-of-care backbone [1]. Despite these advances, long-term survival remains poor overall (5-year OS approximately 30% across all AML), and outcomes are particularly unfavorable in the relapsed/refractory (R/R) setting and in patients with adverse-risk genetics, underscoring the continued need for genotype-directed targeted therapies such as the menin inhibitors reviewed here [1,2].

Over the past decade, the treatment landscape for de novo and relapsed/refractory (R/R) Acute Myeloid Leukemia (AML) has been transformed by the introduction of molecularly targeted therapies [1]. Regulatory approvals of midostaurin, quizartinib, and gilteritinib for FLT3-mutated AML, and enasidenib and ivosidenib for IDH1/IDH2-mutated AML, and venetoclax—a BCL-2 inhibitor now approved in combination with hypomethylating agents across essentially all newly diagnosed AML subtypes ineligible for intensive chemotherapy—have shown that targeting driver oncogenic alterations or key survival pathways in AML is clinically feasible and improves patient outcomes. Whether menin inhibitors can achieve a similar impact remains an important question [2,3].

KMT2A (lysine methyltransferase 2A, also known as MLL1) rearrangements (KMT2Ar) account for about 4–10% of AML cases, 10–15% of acute lymphoblastic leukemia (ALL) cases, and 80% of infant ALL, with a global incidence of around 7000 cases per year [4]. NPM1-mutated AML (mNPM1) is more common, representing about 30% of AML cases worldwide and approximately 20,000 cases annually [5,6]. Despite biological and epidemiological differences, both depend critically on the interaction between the scaffold protein menin (encoded by MEN1) and MLL fusion proteins, or on aberrantly cytoplasmic NPM1, which drives leukemogenic HOX and MEIS1 gene expression [7]. This shared vulnerability provides the molecular rationale for menin inhibition as a unifying therapeutic approach. 

Cytogenetically normal, NPM1-mutated AML—historically considered intermediate-favorable risk—carries a 5-year OS of approximately 50–60% with conventional therapy in the absence of adverse co-mutations (notably FLT3-ITD), while outcomes are substantially worse in the presence of such co-mutations or in the R/R setting [8,9]. The unmet need is therefore substantial, particularly in the R/R setting where, before revumenib, no therapy specifically targeting NPM1-mutated AML or KMT2A-rearranged leukemia had received regulatory approval (patients with co-occurring actionable lesions, such as FLT3 or IDH1/2 mutations, may nevertheless have other approved targeted options).

This is a narrative, non-systematic review; formal systematic-review methodology (e.g., PRISMA, dual independent screening, risk-of-bias assessment) was not applied. The manuscript synthesizes clinical evidence for four menin inhibitors: revumenib (SNDX-5613), ziftomenib (KO-539), bleximenib (JNJ-75276617), and enzomenib (DSP-5336), identified through targeted PubMed/MEDLINE searches and review of recent ASH, EHA, and ASCO conference proceedings, supplemented by regulatory agency communications. It examines their mechanisms, preclinical evidence, clinical efficacy, and safety as monotherapy and in combination, emerging resistance mechanisms, and implications for treatment selection in hematology practice.

## 2. Molecular Rationale: The Menin–MLL Axis

### 2.1. Menin as a Transcriptional Scaffold in Leukemogenesis

Menin is a 610-amino acid nuclear scaffold protein encoded by the tumor suppressor gene MEN1 on chromosome 11q13. Under physiological conditions, menin acts both as a tumor suppressor and as a component of the MLL1/COMPASS (complex of proteins associated with Set1) histone H3K4 methyltransferase complex [10,11]. In the setting of KMT2A rearrangements, however, menin is recruited by the oncogenic MLL fusion protein and becomes an obligate co-activator of transcription of leukemogenic target genes [10,12].

KMT2A rearrangements result from chromosomal translocations juxtaposing the KMT2A gene with more than 80 partner genes, most commonly AFF1 (t(4;11)), MLLT3 (t(9;11)), MLLT10 (t(10;11)), and ELL (t(11;19)) [10]. The resulting fusion proteins lack the carboxy-terminal SET domain responsible for H3K4 methyltransferase activity but retain the amino-terminal menin-binding domain. Menin binding is indispensable: it anchors the MLL fusion protein to specific genomic loci—notably the HOXA cluster and MEIS1—and facilitates aberrant recruitment of the super elongation complex, leading to sustained transcriptional activation and differentiation arrest [12,13].

In mNPM1 AML, the mechanism is distinct but convergent. Mutant NPM1 (most commonly the type A frameshift mutation) inserts a nuclear export signal at the C-terminus of the protein while disrupting its tryptophan-dependent nucleolar-localization residues; the mutant protein is consequently exported to, and retained in, the cytoplasm (hence the designation NPM1c). In this setting, cytoplasmic NPM1c is associated with persistent activation of HOX/MEIS1 transcriptional programs (including HOXA9/A10 and MEIS1) that remain dependent on MLL1–menin-associated chromatin regulation. Menin inhibition suppresses this transcriptional state, although NPM1c itself is not considered a direct menin-binding partner. The precise causal relationship between NPM1c mislocalization, chromatin regulation, MLL1–menin occupancy and menin dependence has not been fully resolved; nonetheless, the HOXA/MEIS1 program is pharmacologically menin-dependent, which provides the molecular rationale for menin inhibitor activity in mNPM1 AML [14,15]. In Npm1c knock-in mouse models, HOX gene upregulation was reversed by Men1 deletion, supporting menin as a non-oncogene addiction in this molecular context [14,15] (Figure 1).

### 2.2. Structural Basis for Menin Inhibition

The menin–MLL interaction is mediated by a well-defined binding groove on the menin surface that accommodates the MLL interaction motif (MIM) of MLL1/fusion proteins. Structural studies have delineated two key domains: MIM-N (a 12-residue peptide containing the critical octapeptide RPPVDFSM) and MIM-C [16,17]. This interaction is tractable to small-molecule disruption, and high-throughput screening campaigns identified initial thienopyrimidine and hydroxyproline scaffolds that were subsequently optimized for potency, selectivity, oral bioavailability, and central nervous system penetration [18].

Critical resistance hotspots—particularly M327, G331, S160, and T349 in the menin-binding groove—have been identified through both structural modeling and clinical observation, thereby informing second-generation compound design [19]. Of note, ziftomenib and bleximenib retain nanomolar activity against common resistance mutations (M327I/V, G331R), whereas revumenib shows reduced binding to T349M [20,21,22]. Table 1 summarizes the key structural and pharmacological features that distinguish the first-generation agent revumenib from the subsequent second-generation compounds ziftomenib, bleximenib, and enzomenib, in place of a schematic chemical structure figure (the exact binding-mode co-crystal structures remain proprietary to the sponsoring companies for several of these agents and are not yet fully disclosed in the public domain).

### 2.3. Preclinical Evidence Supporting Menin Inhibition

Before entering clinical development, the therapeutic rationale for menin inhibition was established through extensive preclinical work. Krivtsov et al. developed VTP-50469, the direct chemical precursor of revumenib, and demonstrated that this compound selectively displaced menin from chromatin in KMT2A-rearranged cell lines (MOLM13, RS4;11) with picomolar biochemical potency (Ki = 104 pM), induced myeloid differentiation and apoptosis, and produced dramatic reductions in leukemic burden in patient-derived xenograft (PDX) models of both KMT2A-rearranged AML and ALL—with a subset of ALL-engrafted mice remaining disease-free for over one year after treatment cessation [23]. Analogous preclinical activity was subsequently confirmed in NPM1-mutant AML models, in which Npm1c knock-in mice developed a HOXA/MEIS1-driven leukemic phenotype that was reversed by genetic Men1 deletion, providing in vivo evidence of menin dependency in this molecularly distinct subset [14,15]. These xenograft and genetically engineered mouse studies were instrumental in selecting the dose ranges and schedules subsequently tested in the first-in-human trials of revumenib, ziftomenib, bleximenib, and enzomenib discussed below, and—together with parallel preclinical work on NUP98-rearranged leukemia discussed in Section 2.4—established the mechanistic rationale that ultimately extended clinical evaluation beyond KMT2Ar and mNPM1 AML.

### 2.4. Beyond KMT2Ar and mNPM1: Nucleoporin (NUP98)-Rearranged Leukemias

Rare AML subtypes driven by chromosomal rearrangements of the nucleoporin gene NUP98—most commonly NUP98::NSD1 and NUP98::KDM5A—represent a third molecular context in which menin inhibition may be active. NUP98 fusion proteins are recruited to HOXA-cluster and MEIS1 target genes through the same MLL1–menin complex exploited by KMT2A fusions, and preclinical studies have shown that primary NUP98-rearranged AML cells and patient-derived xenografts, including those co-harboring FLT3-ITD, are highly sensitive to menin inhibition with revumenib and with earlier tool compounds, with menin blockade inducing differentiation and abrogating leukemic engraftment in vivo [23,24]. Clinically, a limited number of NUP98-rearranged patients enrolled within the broader AUGMENT-101 trial and achieved objective responses, and an isolated case report has described a durable complete remission with a covalent menin inhibitor in relapsed NUP98::NSD1 AML; however, prospective clinical data specifically addressing this population remain sparse, and NUP98-rearranged AML is not currently an approved indication for any menin inhibitor [24]. Given the poor prognosis historically associated with NUP98-rearranged AML, particularly in the presence of FLT3-ITD, dedicated prospective evaluation of menin inhibitors in this subgroup represents an important area for future clinical investigation.

## 3. Clinical Development of Menin Inhibitors

### 3.1. Revumenib (SNDX-5613)

#### 3.1.1. AUGMENT-101: Monotherapy in R/R KMT2Ar and mNPM1 Acute Leukemia

The AUGMENT-101 trial (NCT04065399) is the pivotal international, open-label phase I/II study that established revumenib as the first approved menin inhibitor [25]. Adults and pediatric patients aged 30 days or older with R/R acute leukemia harboring KMT2Ar or NPM1m were enrolled (estimated N = 413). Phase I established the recommended phase II dose (RP2D) based on CYP3A4 inhibitor co-administration: revumenib 163 mg every 12 h with a strong CYP3A4 inhibitor, or 276 mg every 12 h without one, administered in 28-day cycles.

In the pivotal KMT2Ar cohort (Cohorts 2A/B, N = 104), baseline characteristics reflected a heavily pretreated population: 83% AML, 15% ALL, 2% MPAL; 59% had refractory relapse; median 2 prior regimens (range 1–11); 44% had prior allogeneic hematopoietic stem cell transplantation (HSCT). The co-primary endpoints (CR + CRh rate and safety) were met: 22/104 patients (21.2%; 95% CI: 13.8–30.3) achieved CR + CRh, with a CR rate of 12.5% (95% CI: 6.8–20.4). Median duration of CR + CRh was 6.4 months (95% CI: 2.7-NE). These data supported FDA approval of revumenib on 15 November 2024 for adult and pediatric patients aged 1 year and older with R/R acute leukemia with a KMT2A translocation; the indication is not restricted by lineage (the pivotal cohort comprised AML, ALL and MPAL), and patients with KMT2A partial tandem duplication were excluded [25,26].

In Cohort 2C (mNPM1 AML, N = 64 adult efficacy population), responses occurred rapidly [27]: CR + CRh was achieved in 15 patients (23.4%), with a median time to CR + CRh of 2.76 months (range 1.80–8.80) and median duration of 4.7 months (range 1.2–8.2 measured from the date of first CR/CRh to progression, relapse, or death, irrespective of the number of revumenib cycles received—patients could, and often did, continue treatment for further cycles beyond that landmark, consistent with the continuous 28-day-cycle dosing schedule used in AUGMENT-101). The overall response rate (ORR) was 30/64 (46.9%), with a median time to first response of 1.84 months (range 0.90–4.60) and a median duration of overall response of 4.4 months (range 1.2–5.6 again calculated from first response regardless of cycle number). Regulatory evaluation of revumenib for R/R mNPM1 AML, which was ongoing at the time of initial submission, concluded with FDA approval on 24 October 2025 for adult and pediatric patients aged 1 year and older with R/R AML with a susceptible NPM1 mutation and no satisfactory alternative treatment options, based on an updated efficacy analysis (CR + CRh rate 23.1%, 95% CI 13.5–35.2, in the FDA review [28]; 23.4% and ORR 46.9% in the published adult efficacy population [27]).

#### 3.1.2. Combination Approaches with Revumenib

The Beat AML trial substudy (NCT03013998) evaluated revumenib combined with venetoclax and azacitidine (VEN/AZA) in patients over 60 years with newly diagnosed AML and mNPM1 or KMT2A who were ineligible for intensive chemotherapy (N = 43) [29,30]. Dose levels 1 (DL1: 113 mg Q12H, n = 21) and 2 (DL2: 163 mg Q12H, n = 22) were explored. Only one hematologic DLT (grade 4 thrombocytopenia) occurred in DL1; no maximum tolerated dose was identified. Grade ≥ 3 non-hematologic TEAEs occurred in 26% (febrile neutropenia), and differentiation syndrome (DS) occurred in 18.6% (grade ≥ 3: 4.7%). No grade 4 DS or QTcF prolongation was observed, and no patient discontinued revumenib due to DS or QTcF prolongation [31].

Efficacy results were encouraging: the overall response rate (CR/CRh/CRi/MLFS) was 88.4% across both dose levels, with 67.4% achieving complete remission (CR). In the NPM1-mutant subgroup (n = 34), CR was 64.7%; in the KMT2Ar subgroup (n = 9), CR was 77.8%. Notably, 84% of evaluable patients achieved remission within the first cycle, 100% of evaluable responders achieved flow MRD-negativity (sensitivity 0.02%), and 31% achieved NPM1m NGS-negative remission (sensitivity 0.005%)—a notable depth of response for this frail population, although these single-arm data cannot be compared directly with VEN/AZA alone [31].

The SAVE Phase I/II trial (MD Anderson Cancer Center, ASH 2024) explored revumenib combined with an all-oral regimen of venetoclax and decitabine/cedazuridine in pediatric and adult R/R AML or MPAL with mNPM1, KMT2Ar, or NUP98r (N = 33). The ORR was 82%, with CR/CRh in 48%. MRD negativity by multiparameter flow cytometry was achieved in 65% of responders. The combination was generally well tolerated. The trial is now enrolling newly diagnosed patients with a menin inhibitor-based all-oral regimen, highlighting the therapeutic potential of this approach [30].

### 3.2. Ziftomenib (KO-539)

#### 3.2.1. KOMET-001: Monotherapy in R/R mNPM1 AML

Ziftomenib, a selective oral menin inhibitor administered once daily, was evaluated in KOMET-001 (NCT04067336), an international, open-label, multicohort, first-in-human phase I/II trial. The registration-enabling phase II enrolled R/R mNPM1 AML patients at 600 mg QD (N = 92 phase II; N = 112 pooled phase Ib/II) [8,32,33].

The primary phase II endpoint (CR/CRh) was met, with a CR/CRh rate of 23% (21/92), statistically superior to the historical control rate of 12% (*p* = 0.0058), and an overall response rate of 33% (30/92). In the pooled phase Ib/II analysis (N = 112), CR/CRh was 25%, and ORR was 35%. Median time to CR/CRh was 2.8 months, and median time to objective response was 1.9 months. MRD negativity was achieved in 63% (12/19) of evaluable CR/CRh responders in the phase II study and in 65% (17/26) in the pooled analysis. Median OS in the pooled population was 6.1 months (95% CI: 3.8–8.4), with a longer observed OS in responders (median 16.4 months, 95% CI: 9.6-NE) and non-responders (median 3.5 months, 95% CI: 2.4–4.0) [32], an observational comparison subject to responder bias. Based on these data, ziftomenib (brand name Komzifti) received FDA approval on 13 November 2025, for adult patients with R/R AML with a susceptible NPM1 mutation and no satisfactory alternative treatment options; in the FDA-evaluated efficacy population (N = 112) the CR + CRh rate was 21.4% (95% CI: 14.2–30.2), which differs from the 25% reported in the published pooled analysis, the FDA figure being the regulatory reference. Komzifti is administered once daily and carries a boxed warning for differentiation syndrome, but not for QTc prolongation, which is listed as a warning and precaution [34,35].

#### 3.2.2. KOMET-007: Ziftomenib in Combination with Chemotherapy

KOMET-007 (NCT05735184) is a phase I multicohort study evaluating ziftomenib combined with 7 + 3 (cytarabine/daunorubicin) or venetoclax/azacitidine in mNPM1 or KMT2Ar AML [8,29,36,37,38]. In the newly diagnosed, intensive chemotherapy-eligible cohort at 600 mg (NPM1: n = 49; KMT2Ar: n = 33), ziftomenib was initiated on day 8 of induction and continued thereafter. After a median follow-up of 24.9 weeks in the NPM1 cohort, 96% (47/49) of patients remained alive on study or in long-term follow-up, with median OS not reached. In the KMT2Ar cohort (median follow-up 15.7 weeks), the CR rate was 85% (28/33), and the median OS was also not reached. Grade ≥ 3 TEAEs matched the expected toxicity profile of 7 + 3 chemotherapy: febrile neutropenia occurred in 60% of patients, with no unexpected toxicity from ziftomenib addition [37].

In the R/R Ven/Aza cohort (KOMET-007, ASH 2024, N = 49 mNPM1 + N = 27 KMT2Ar at doses 200–600 mg), the overall response rate was 68% in mNPM1 (15/22 at all doses combined) and 33% in KMT2Ar. The 6-month OS was 77% in mNPM1 and 43% in KMT2Ar patients, which are encouraging but cannot be formally compared with historical benchmarks in this non-randomized setting [36].

### 3.3. Bleximenib (JNJ-75276617)

#### 3.3.1. cAMeLot-1: Monotherapy

Bleximenib is an oral menin inhibitor notable for its structural activity against resistance mutations M327I/V and T349M, albeit with reduced potency versus wild-type (IC50 1.5 nM, 20.1 nM, and 0.6 nM for M327I, T349M, and WT, respectively; Ki 0.4, 3.8, and 0.02 nM) [20,39]. The cAMeLot-1 trial (N = 146 dosed) established an RP2D of 100 mg BID orally, with a 50 mg BID step-up dose for 2 weeks, in patients with R/R KMT2Ar, mNPM1, or NUP98/NUP214-altered acute leukemia [38,40].

At the optimal dose (90/100 mg BID), the ORR was 47.6% (10/21), with CRc (CR/CRh/CRi) in 38.1% and CR/CRh in 33.3%. At the 150 mg BID level, ORR was 55.0% with CRc 40.0%. The response by molecular subgroup at the 90/100 mg BID level was KMT2Ar (n = 9): cCR 44.4%, CR/CRh 33.3%; mNPM1 (n = 12): cCR 33.3%, CR/CRh 33.3%. Median time to first response was 1.4 months (range 0.9–4.7) at the RP2D level. Median duration of CR/CRh across all dosed patients was 6 months (95% CI: 1.9-NE). Safety was favorable at RP2D: AE-related discontinuations were 6.5%, versus more frequent dose modifications at 150 mg BID [38,40].

#### 3.3.2. Bleximenib + VEN/AZA in KMT2Ar and mNPM1 AML

An international, open-label, phase I trial (NCT05453903, EHA 2025) evaluated bleximenib combined with VEN + AZA in R/R AML (Cohort A3, n = 85) and newly diagnosed AML (Cohort B1, n = 40 targeted) [41]. At the recommended dose of 100 mg BID in combination, no DLTs were identified. Among R/R patients at 100 mg BID, the ORR was 70.0% in KMT2Ar (n = 10) and 91.7% in mNPM1 (n = 12); cCR was 50.0% and 66.7%, respectively. In the newly diagnosed cohort, ORR was 75.0% in KMT2Ar (n = 4) and 93.8% in mNPM1 (n = 16), with cCR rates of 75.0% each—results that cannot be compared directly with those of VEN/AZA alone because the data are non-randomized and the cohorts small. In an exploratory analysis, no acquired MEN1 resistance mutations were found in 19 evaluable R/R patients; given the very small number of patients and the dependence on assay sensitivity and sampling time, this observation is insufficient to establish that VEN/AZA prevents MEN1-mediated resistance [41]. This early combination program provided the foundation for the confirmatory phase III cAMeLot-2 trial described in Section 7.3.

### 3.4. Enzomenib (DSP-5336)

Enzomenib is an oral menin inhibitor evaluated in a phase I/II study in R/R acute leukemia with KMT2Ar or mNPM1 (N = 84 as of ASH 2024) [42]. No DLTs were observed at any dose tested (200 and 300 mg BID), and no discontinuations due to drug-related adverse events occurred [42]. At 300 mg BID in KMT2Ar (n = 15), the ORR was 73.3%, composite CR (CR/CRh/CRi) 53.3%, and CR + CRh 40.0%; at 300 mg BID in mNPM1 (n = 7), ORR was 57.1%, composite CR 42.9%, and CR + CRh 42.9%. Median time to first objective response was 1.0 months for KMT2Ar and 1.9 months for mNPM1. Median duration of response for KMT2Ar was not reached, at 7.0 months, and 34% and 12% of patients, respectively, proceeded to allogeneic HSCT for mNPM1. Differentiation syndrome occurred in 10.7% (9/84) with no mortality or permanent discontinuations; notably, no DS prophylaxis or step-up dosing was used with enzomenib [42].

## 4. Class-Specific Toxicities and Management

### 4.1. Differentiation Syndrome

Differentiation syndrome (DS) is the most clinically significant class-specific adverse event of menin inhibitors, sharing pathophysiology with the DS observed with IDH inhibitors and ATRA in acute promyelocytic leukemia. It results from the rapid differentiation of leukemic cells following suppression of the HOX/MEIS1 program, leading to a systemic inflammatory response mediated by cytokine release from differentiating myeloid cells [43].

Reported DS rates vary by agent, cohort, data cut and DS-prophylaxis strategy and should not be pooled or compared directly across trials. For revumenib, DS occurred in 28% (grade ≥ 3: 16%) of the AUGMENT-101 KMT2Ar efficacy population [26] and in 25% (grade 3–4: 12%) of the 241 patients in the pooled safety population of the FDA label [44]. For ziftomenib, the FDA label (R/R mNPM1 AML, N = 112) is used as the single primary source: DS occurred in 26% of patients (grade ≥ 3: 13%, including two fatal cases) [35]; the published KOMET-001 phase II report (N = 92) gave comparable figures (25% all-grade; grade 3 in 15%) [32], whereas lower earlier estimates (approximately 12–15% all-grade; 5% grade ≥ 3 in phase Ib cohorts) reflect different cohorts and data cuts, are reported separately in Table 2 and are not combined with the registration-enabling estimates [33]. For enzomenib, DS occurred in 10.7% (9/84), with no grade ≥ 3 events reported to date [42]. No source reporting an absolute 0% DS rate for any agent was identified. DS prophylaxis and monitoring practices differ across trials and may partly explain differences in severe DS rates between agents. Ziftomenib had grade 4/5 DS as DLTs in phase I. Bleximenib had a grade 5 DS event as a DLT at 150 mg BID in phase I, but no grade 4/5 events at the RP2D of 100 mg BID. Enzomenib reported no grade 3+ DS and no DS-related deaths or discontinuations without prophylaxis [33,40,42].

Clinical recognition of DS requires vigilance for unexplained fever, hypoxia, pulmonary infiltrates, peripheral edema, pleural or pericardial effusion, and renal insufficiency. Management involves immediate systemic corticosteroids (dexamethasone 10 mg IV every 12 h) and temporary drug interruption in severe cases. Hydroxyurea may be considered for hyperleukocytosis accompanying DS [43].

### 4.2. QTc Prolongation

QTc prolongation is an important toxicity distinguishing revumenib from the other agents in this class. In AUGMENT-101, QTc prolongation of any grade occurred in 26% of patients and grade 3+ in 14% [26]. Revumenib’s FDA label includes a boxed warning for QTc prolongation, and co-administration requires careful attention to CYP3A4 interactions (revumenib itself is a moderate CYP3A4 inhibitor [26,27,44]). In the published reports, ziftomenib, bleximenib, and enzomenib were not associated with clinically significant QTc prolongation at recommended doses [32,40,42]; however, the ziftomenib FDA label lists QTc prolongation as a warning and precaution (12% any grade; 8% grade 3 among 112 patients; baseline ECG and electrolyte correction required) [35], so the apparent difference from revumenib, whose label carries a boxed warning, should be interpreted cautiously and cannot be generalized across agents or trials. Drug–drug interactions with azole antifungals—routinely used for antifungal prophylaxis during intensive AML therapy—deserve specific comment, as this class illustrates the CYP3A4-related management differences across menin inhibitors. Revumenib is a CYP3A4 substrate, and its dosing is explicitly stratified by co-administration of a strong CYP3A4-inhibiting azole (itraconazole, ketoconazole, posaconazole, voriconazole): the approved starting dose is reduced from 270 mg (160 mg/m^2^ for patients <40 kg) to 160 mg (95 mg/m^2^) every 12 h (labeled doses; the trial publications report 276 mg and 163 mg) when a strong azole is co-administered, with dose re-escalation recommended after discontinuation of the azole once at least five half-lives have elapsed [44]. Because posaconazole, voriconazole, and itraconazole are all strong or moderate CYP3A4 inhibitors and are frequently used as first-line antifungal prophylaxis in AML, this interaction has direct practical relevance and requires close coordination between hematologists and antimicrobial stewardship teams. Ziftomenib is primarily metabolized by CYP3A; its FDA label states that concomitant strong or moderate CYP3A inhibitors increase exposure and advises more frequent monitoring for adverse reactions, and published analyses did not identify a need for routine dose adjustment with azoles [35], while data on bleximenib and enzomenib in combination with azoles remain more limited; the enzomenib phase I/II study specifically incorporated parallel dosing arms with and without concomitant antifungal azoles to characterize this interaction prospectively [42,43,44].

### 4.3. Hematologic Toxicities

Cytopenias—including febrile neutropenia, thrombocytopenia, and anemia—are common across all menin inhibitors, largely reflecting the expected toxicity of AML itself and background chemotherapy in combination regimens. In monotherapy phase II trials, grade 3+ febrile neutropenia rates range from 22% (ziftomenib) to 38% (revumenib); grade 3+ thrombocytopenia rates range from 7% (ziftomenib) to 31% (bleximenib). The apparently lower myelosuppression of ziftomenib monotherapy in cross-trial observation may relate to dosing, binding kinetics or patient selection, and cannot be attributed to the drug itself without comparative data [43]. With respect to severity and duration specifically: in AUGMENT-101, grade ≥ 3 neutropenia and thrombocytopenia were each reported in more than half of revumenib-monotherapy patients, with a median time to platelet and neutrophil recovery (to ≥50,000/µL and ≥1000/µL respectively, among responders) of approximately 1–2 months from response; in combination regimens incorporating venetoclax/azacitidine or intensive chemotherapy (Beat AML, KOMET-007 ND cohort), grade ≥ 3 cytopenias were near-universal and more prolonged, consistent with the added myelosuppressive contribution of the chemotherapy backbone rather than the menin inhibitor per se, and recovery was generally assessed and managed using cycle-delay and growth-factor-support algorithms similar to those used with venetoclax-based regimens outside this drug class. These duration data, where available by agent and regimen, are incorporated into Table 2 (monotherapy) and Table 3 (combination regimens) below [26,31,37,43].

## 5. Resistance Mechanisms

### 5.1. Acquired MEN1 Mutations

The primary mechanism of acquired resistance to menin inhibitors identified to date involves somatic point mutations in the MEN1 gene that directly impair drug binding to the menin–MLL interaction interface [19]. MEN1 mutations were first described as mediators of clinical resistance by Perner et al. [19]; in AUGMENT-101, such mutations were identified (M327I, M327V, G331R, G331D, T349M) in 38.7% of patients who received more than two cycles of revumenib and subsequently progressed. These mutations emerge rapidly—typically by cycle 3, day 1—and can be polyclonal, with multiple resistance mutations occurring simultaneously in individual patients [26,27].

Structural analysis reveals that M327 and G331 reside within the menin-binding pocket, in direct contact with revumenib, while T349M induces an allosteric conformational change, reducing drug affinity. Notably, bleximenib—designed with these resistance mutations in mind—retains nanomolar activity against M327I/V (Ki 0.4 nM) and reduced but meaningful activity against T349M (Ki 3.8 nM) [20,39]. This raises the hypothesis that bleximenib or other second-generation agents might be effective post-revumenib, an approach currently under investigation [20,45].

### 5.2. Additional Resistance Mechanisms

Beyond MEN1 mutations, other emerging resistance mechanisms include: (1) RAS/MAPK pathway activating mutations (KRAS G12D/V, NRAS Q61K) that bypass menin dependency; (2) FLT3 internal tandem duplications emerging at relapse; (3) chromatin remodeling alterations; and (4) clonal evolution leading to loss of the target allele or oncogene switching [46,47].

## 6. Treatment Selection and Clinical Considerations

### 6.1. Molecular Eligibility and Testing

Accurate molecular characterization is a prerequisite for eligibility for menin inhibitors. KMT2A rearrangements must be detected by conventional cytogenetics and/or FISH—critically, KMT2A partial tandem duplications (KMT2A-PTD) do not appear to confer sensitivity to menin inhibitors and should not be conflated with KMT2A rearrangements (fusions). NPM1 mutations are reliably detected by PCR or next-generation sequencing (NGS); the mutation type (predominantly type A) does not alter menin inhibitor sensitivity. Neither FDA announcement names a specific companion diagnostic; a susceptible NPM1 mutation was confirmed in the pivotal cohorts by NGS or PCR of the last exon of NPM1 [28]. Molecular retesting at relapse is advisable given the dynamic nature of leukemia and the risk of clonal evolution that may introduce or eliminate relevant alterations [48,49,50,51]; clonal evolution at relapse is described in [46,47].

### 6.2. Impact of Prior Therapy on Response

Prior therapy history significantly modulates response to menin inhibitor monotherapy. In AUGMENT-101, prior venetoclax exposure and prior FLT3 inhibition were associated with lower CR/CRh rates to revumenib monotherapy. Conversely, co-mutations in IDH1 or IDH2 were associated with higher CR/CRh rates. These observations are hypothesis-generating; they underscore the importance of individualized risk-benefit assessment and, if confirmed, would support evaluating menin inhibitors earlier, before the acquisition of cross-resistance [29].

### 6.3. Treatment Goals: The Critical Role of Allotransplantation

With monotherapy response durations of only 4–6 months (median CR/CRh duration), allogeneic HSCT following any objective response must be strongly considered in transplant-eligible R/R AML patients—a recommendation that spans all menin inhibitors reviewed. The bridge-to-transplant paradigm is explicitly embedded in clinical trial designs and is supported by outcomes data showing durable remissions in patients who proceed to allotransplantation. In the KOMET-007 newly diagnosed NPM1 cohort, 96% of patients remained alive on study, and in the AUGMENT-101 mNPM1 cohort, MRD negativity was achieved in the majority of responders, potentially representing an optimal transplant opportunity [8,27].

### 6.4. Positioning in the Treatment Algorithm

Currently, revumenib is approved for two indications: R/R acute leukemia with a KMT2A translocation (FDA, 15 November 2024; adult and pediatric patients aged 1 year and older; not restricted by lineage, the pivotal cohort comprising AML, ALL and MPAL) and, following an expanded approval, R/R AML with a susceptible NPM1 mutation (FDA, 24 October 2025; adult and pediatric patients aged 1 year and older with no satisfactory alternative treatment options) [25,28]. Ziftomenib (Komzifti) received FDA approval on 13 November 2025, for adults with R/R AML with a susceptible NPM1 mutation and no satisfactory alternative treatment options, making it the second approved menin inhibitor; unlike revumenib, it is administered once daily [34,35]. Bleximenib and enzomenib remain investigational, with regulatory submissions not yet completed at the time of this revision. Both agents should be considered within multidisciplinary team discussions for appropriate R/R patients, noting that revumenib and ziftomenib have not been directly compared in a head-to-head trial, and choice between them in mNPM1 AML may reasonably be guided by dosing convenience (QD vs. BID), QTc/cardiac risk profile, and azole co-medication needs rather than by a demonstrated efficacy difference. Because patients with KMT2Ar or mNPM1 disease may harbor co-occurring mutations (e.g., FLT3 or IDH1/2) that offer other approved targeted options, menin inhibitors should not be regarded as the only, or necessarily the first, targeted option for every patient, and treatment selection should be individualized.

For newly diagnosed patients, combination strategies incorporating menin inhibitors with VEN/AZA (for HMA-eligible patients) or with intensive induction chemotherapy (7 + 3) represent the next therapeutic frontier. Early data from Beat AML (revumenib + VEN/AZA) and KOMET-007 (ziftomenib + 7 + 3) are encouraging, and randomized phase III trials will be essential to definitively establish the benefit over standard-of-care backbones.

## 7. Comparative Summary and Emerging Agents

To address overlap between the comparative and trial-listing tables of the original submission and to allow a clearer comparison by disease subset and treatment setting, this section now presents three non-overlapping tables: Table 2 summarizes monotherapy trials, separated by molecular subgroup (KMT2Ar vs. mNPM1); Table 3 summarizes combination-regimen trials; and Table 4 summarizes ongoing or planned randomized controlled trials (RCTs), none of which have yet reported results. The agents differ in: dosing schedule (revumenib and bleximenib BID vs. ziftomenib QD); CYP3A4/azole interaction (most relevant for revumenib); QTc prolongation risk (boxed warning for revumenib; a warning and precaution for ziftomenib); preclinical activity against MEN1 resistance variants (which differs among agents); and regulatory status (revumenib and ziftomenib now both approved for their respective indications, as detailed in Section 6.4). Several additional menin inhibitors are in earlier clinical development, including BMF-219 (an oral covalent inhibitor with a potentially distinct resistance profile), HH2853, BN104 [52], DS-1594 [53], and various preclinical programs targeting the menin–MLL interface through alternative binding modes. The therapeutic landscape is expected to expand considerably over the next five years.

### 7.1. Monotherapy Trials by Molecular Subgroup

Table 2 reports comparative efficacy and safety exclusively for monotherapy trials, with KMT2A-rearranged and NPM1-mutated cohorts of the same trial listed as separate rows to avoid conflating two biologically distinct populations, and includes enrolled/evaluable patient numbers, trial sponsor, and hematologic-toxicity duration data as requested.

### 7.2. Combination-Regimen Trials

Table 3 is restricted to combination trials (menin inhibitor plus venetoclax/hypomethylating agent or intensive chemotherapy).

### 7.3. Ongoing/Planned Randomized Controlled Trials

Table 4 lists the randomized controlled trials of menin inhibitors currently recruiting or planned. To date, no randomized trial of a menin inhibitor has reported results, and no published RCT has yet demonstrated a complete-remission or overall-survival advantage for any menin inhibitor, either as monotherapy or in combination—a limitation now explicitly stated in Section 8 (Discussion) and Section 9 (Conclusions).

## 8. Discussion

Menin inhibitors represent one of the most mechanistically coherent advances in AML therapy since the introduction of IDH inhibitors, and the clinical data reviewed here are broadly consistent with a trajectory analogous to the paradigm shifts established by FLT3 and IDH-targeted therapy; however, as detailed below, this comparison should be tempered by the current absence of randomized, comparative evidence [1,54,55,56].

Several observations from the current data landscape are particularly instructive for the expert hematologist. First, the depth of response achievable with menin inhibitor-based combinations in newly diagnosed AML is notable. The 100% flow MRD-negativity rate observed in the Beat AML revumenib + VEN/AZA substudy—in a population aged 60 years or older deemed unfit for intensive induction—is notable, but cannot be compared directly with the results of VEN/AZA alone given differences in patient populations, MRD assay sensitivity and the non-randomized design [31,54]. If durable, this depth of response could meaningfully alter long-term survival in these patients, although this claim requires confirmation in the randomized cAMeLot-2 and EVOLVE II trials (Table 4), since single-arm MRD data, however encouraging, cannot establish a survival benefit on their own.

Second, the resistance landscape deserves attention. The identification of MEN1 mutations as the primary resistance mechanism creates both a diagnostic imperative and a therapeutic opportunity. Liquid biopsy-based monitoring of MEN1 mutation emergence should be integrated into clinical management of patients on menin inhibitors, as early detection may allow timely transition to salvage strategies—including second-generation inhibitors with enhanced resistance mutation coverage. [45,47]. This also relates directly to the dosing rationale discussed in Section 4.2: because M327 and G331 sit within the direct drug-binding pocket contacted by revumenib, whereas T349M acts allosterically, it is mechanistically the mutation-binding-site geometry—rather than necessarily an excessively high applied dose—that appears to underlie revumenib’s susceptibility to on-target resistance; no preclinical or clinical dose-de-escalation study has yet formally tested whether a lower revumenib dose would preserve efficacy while reducing toxicity, and this remains an open question for future investigation [19,27,45]. In the small exploratory bleximenib + VEN/AZA cohort of R/R patients (19 evaluable patients), no acquired MEN1 resistance mutations were detected; this observation depends on assay sensitivity and sampling time, is insufficient to establish that VEN/AZA prevents MEN1-mediated resistance, and would need validation in larger series before informing the design of combination strategies [41].

Third, the differentiation syndrome profile across agents is heterogeneous and reflects both pharmacological differences and clinical trial management protocols. The absence of DS-related mortality with enzomenib, without DS prophylaxis, derives from a small early-phase dataset and cannot be interpreted as an advantage of this agent over other menin inhibitors; it may reflect patient selection, dose, follow-up or management differences [42,43]. Standardized DS management protocols and, ideally, prospective DS biomarker studies would greatly facilitate cross-trial comparisons and clinical decision-making.

Fourth, the question of sequencing and combination remains open. Whether menin inhibitors are best employed as monotherapy bridges to transplant, as combinations with HMA/BCL-2 agents for transplant-ineligible patients, or as components of intensive induction regimens for fit newly diagnosed patients will be answered by ongoing and planned randomized trials (Table 4) [54,55]. The data reviewed here suggest all three strategies have merit in specific patient populations, and rational biomarker-guided selection will be critical. Fifth, and centrally, no randomized controlled trial has yet demonstrated a complete-remission or overall-survival advantage for a menin inhibitor, either as monotherapy or in combination, over standard of care, in any AML setting reviewed here. All efficacy data summarized in Table 2 and Table 3 derive from single-arm phase I/II studies, and the encouraging response rates and MRD-negativity figures—while clinically meaningful and the basis for regulatory approval in the R/R setting—do not by themselves establish superiority over existing regimens in a comparative sense. The three randomized trials listed in Table 4 (EVOLVE II, cAMeLot-2, and the randomized expansion of KOMET-007) are designed to address this gap directly, and their results, expected over the next several years, will be essential in determining whether the response and MRD benefits observed to date translate into improved event-free and overall survival. This caveat does not diminish the value of menin inhibitors as genotype-directed options for R/R KMT2Ar and mNPM1 AML, a setting in which no therapy specifically targeting these lesions had previously been approved (although co-occurring mutations, e.g., in FLT3 or IDH1/2, may offer other approved targeted options), but it should temper enthusiasm regarding their role in the frontline setting until randomized data mature.

Sixth, regarding drug-class interactions relevant to routine practice: because azole antifungal prophylaxis is standard of care during intensive AML therapy and post-transplant, and because revumenib requires azole-stratified dosing (Section 4.2), close coordination between the treating hematology team and antimicrobial stewardship or transplant infectious-disease services is warranted whenever revumenib is initiated or an azole is added or discontinued during therapy, to avoid inadvertent under- or over-dosing [44].

### Strengths and Limitations of This Review

This review has several strengths: it synthesizes data across all four clinically advanced menin inhibitors within a single, comparative framework organized by molecular subgroup and treatment setting (monotherapy vs. combination); it incorporates the most recent regulatory developments, including the October 2025 and November 2025 FDA approvals of revumenib and ziftomenib for mNPM1 AML, which post-date several previously published reviews on this topic; and it explicitly addresses ongoing randomized trials and drug-interaction considerations (notably with azole antifungals) that are of direct, practical relevance to treating hematologists but have received comparatively limited attention in prior reviews. Compared with other recent narrative reviews and scoping reviews of menin inhibitors in the literature, including a recent scoping review of safety and efficacy and a general overview of menin inhibition in acute leukemia, the present manuscript places specific emphasis on cross-agent comparison of structural/pharmacological features (Table 1), on the distinction between monotherapy and combination data (Table 2 and Table 3), and on the current absence of randomized comparative evidence (Section 8, points five and six) [43,57].

This review also has important limitations, several of which were highlighted during peer review. First, as noted in Section Overview of Acute Myeloid Leukemia, this is a narrative rather than a systematic review: literature identification relied on targeted PubMed/MEDLINE searches and review of recent major hematology conference proceedings rather than a pre-registered systematic search protocol, dual independent screening, or formal risk-of-bias assessment; consequently, publication and selection bias cannot be excluded, and the review should be interpreted as an expert synthesis rather than a systematic evidence synthesis. Second, cross-trial comparisons of efficacy and safety (Table 2, Table 3 and Table 4) are subject to the well-recognized limitations of indirect comparison across non-randomized, differently designed studies with heterogeneous patient populations, prior-therapy exposure, follow-up duration, response definitions, denominators and data cuts (see footnotes to Table 2 and Table 3); apparent differences between agents (for example, in DS or QTc rates) may partly reflect differences in trial design, dose-finding methodology, and DS-prophylaxis protocols rather than true pharmacological differences. Third, much of the combination-therapy data reviewed here derive from early-phase, non-randomized studies with modest sample sizes (Table 3), and, as discussed in Section 8, no randomized trial data are yet available to confirm a survival benefit. Fourth, given the rapid pace of regulatory and clinical developments in this field, some of the data presented—particularly regarding ongoing trials in Table 4 and evolving regulatory status—should be considered a snapshot as of the time of this revision and may require updating as further results and approvals emerge.

## 9. Conclusions

Menin inhibitors are establishing themselves as a clinically important class of molecularly targeted AML therapy, with a growing—though still evolving—evidence base. Revumenib was the first approved agent in this class and has since received a second FDA approval for R/R mNPM1 AML, and ziftomenib has followed shortly thereafter with its own approval (as Komzifti) for the same indication. Bleximenib and enzomenib introduce structural and pharmacological innovations that may address key limitations of first-generation compounds—such as MEN1 resistance mutations and QTc prolongation; although these potential advantages remain to be confirmed clinically, the agents remain investigational and have not yet completed regulatory review. Combination regimens incorporating menin inhibitors with venetoclax/azacitidine or intensive chemotherapy have shown high response rates in early-phase studies that cannot be compared directly with historical benchmarks, with MRD negativity emerging as a promising, though not yet validated, clinically meaningful endpoint pending confirmation in randomized trials. As emphasized, no randomized controlled trial has yet reported a complete-remission or overall-survival advantage for any menin inhibitor, and much of the evidence for combination regimens in particular remains derived from early-phase, non-randomized studies; the ongoing randomized trials summarized in Table 4 will be essential in establishing the definitive role of these agents across the AML treatment continuum, from first-line therapy in newly diagnosed patients to salvage approaches in the R/R setting, where they represent genotype-directed options for patients with KMT2Ar or mNPM1 disease, although co-occurring mutations (e.g., FLT3 or IDH1/2) may offer other approved targeted options that should be considered.

## Figures and Tables

**Figure 1:**
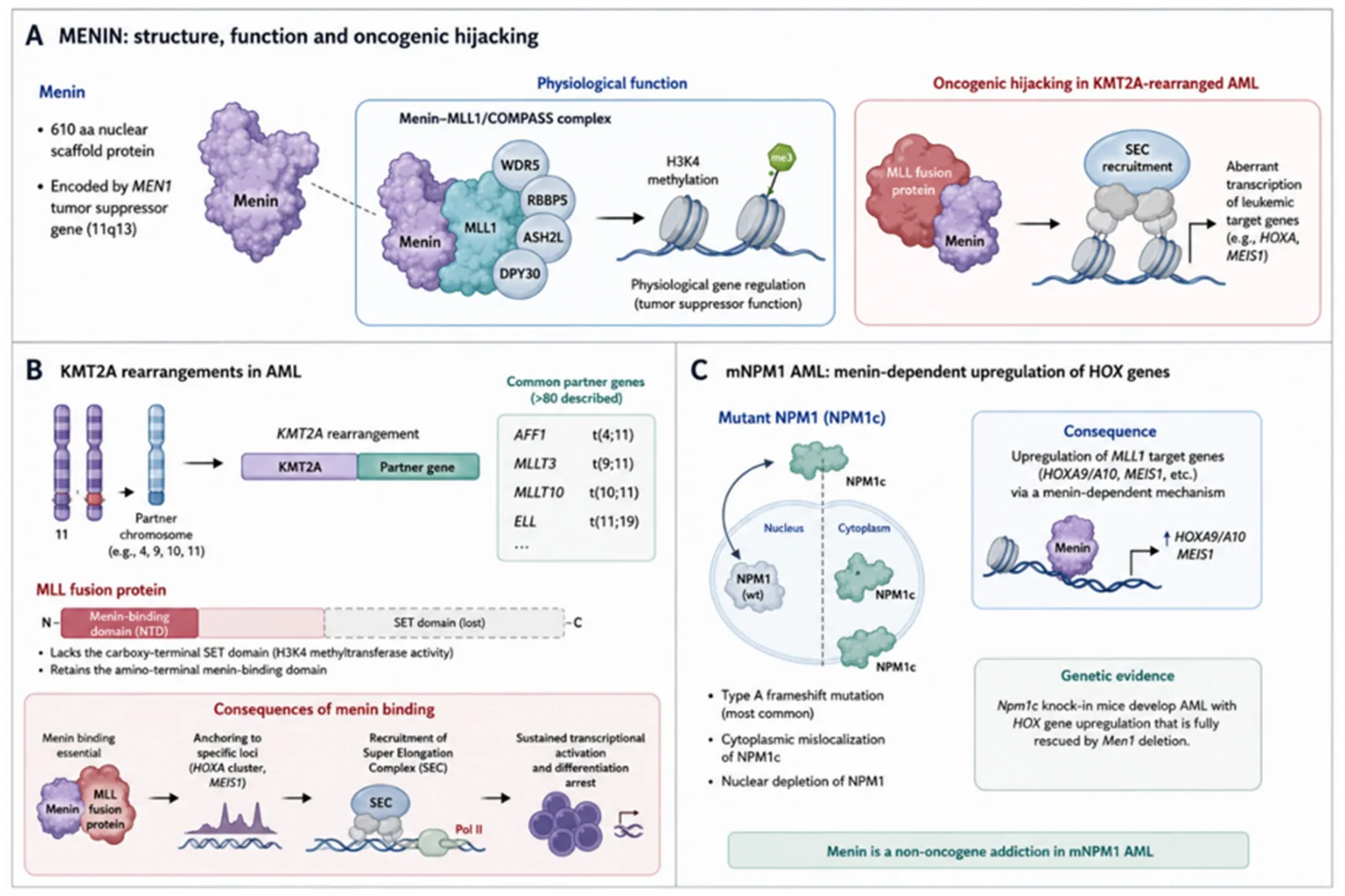
Menin-dependent leukemogenic pathways in AML. (**A**) Menin: physiological function and oncogenic hijacking. Menin, a 610-aa nuclear scaffold protein encoded by *MEN1* (11q13), is a component of the MLL1/COMPASS-like H3K4-methyltransferase complex (WDR5, RBBP5, ASH2L, DPY30) and contributes to transient, developmentally appropriate expression of *HOXA* and *MEIS1* during normal myelopoiesis; it also acts as a tumour suppressor in endocrine tissues. In KMT2A-rearranged AML, this scaffolding role is hijacked: menin tethers the MLL fusion protein to chromatin and enables SEC recruitment, driving aberrant transcription of *HOXA* and *MEIS1*. (**B**) KMT2A rearrangements. Translocation of 11q23 with one of >80 partners (e.g., *AFF1*, *MLLT3*, *MLLT10*, *ELL*) yields a fusion protein that loses the C-terminal SET domain but retains the N-terminal menin-binding domain. Menin anchors the fusion at the *HOXA* cluster and *MEIS1*, enabling SEC and Pol II recruitment, sustained transcription and differentiation arrest. (**C**) mNPM1 AML—convergent mechanism. A type A frameshift generates cytoplasmically mislocalised NPM1c with nuclear NPM1 depletion, associated with menin-dependent upregulation of *HOXA9/A10* and *MEIS1*. NPM1c is not a direct menin-binding partner and the mechanistic link is incompletely defined, but *Npm1c* knock-in mice develop AML with *HOX* upregulation fully rescued by *Men1* deletion—identifying menin as a non-oncogene addiction.

**Table 1:** Comparative structural and pharmacological features distinguishing revumenib from second-generation menin inhibitors. WT, wild-type.

Feature	Revumenib (1st-gen)	Ziftomenib	Bleximenib	Enzomenib
Chemical scaffold	Piperidine/pyrimidine (VTP-50469 derivative)	Macrocyclic pyrimidine	Spirocyclic pyrimidine	Pyrimidine-based, distinct chemotype
Dosing schedule	Twice daily (BID)	Once daily (QD)	Twice daily (BID)	Twice daily (BID)
CYP3A4 interaction	Substrate and moderate inhibitor; dose adjustment required with strong azoles	CYP3A substrate; strong/moderate CYP3A inhibitors increase exposure (closer monitoring advised per FDA label)	Under characterization	Under characterization; azole-stratified dosing arms used in Phase I
Activity vs M327I/V	Reduced	Retained (nanomolar)	Retained (nanomolar)	Retained
Activity vs T349M	Reduced (allosteric escape)	Retained	Retained (reduced potency vs. WT)	Not fully characterized
QTc liability	Boxed warning	Lower signal in published reports; QTc prolongation 12% (grade ≥ 3: 8%) per FDA label (warning/precaution)	Minimal at RP2D	Minimal reported

**Table 2:** Comparative efficacy and safety data for menin inhibitors as monotherapy in relapsed/refractory AML, presented separately by molecular subgroup. Abbreviations: CR, complete remission; CRh, complete remission with partial hematologic recovery; DoR, duration of response; DS, differentiation syndrome; mo, months; NR, not reached; ORR, overall response rate; Ph, phase; RP2D, recommended phase 2 dose. Notes: values are not directly comparable across rows because denominators and data cuts differ. CR + CRh, ORR and DoR refer to the efficacy-evaluable population shown in the N column, whereas QTc and DS rates refer to the safety population of the cited source: revumenib KMT2Ar, AUGMENT-101 KMT2Ar cohort [26]; revumenib mNPM1, pooled safety population of the FDA label (N = 241) [44]; ziftomenib mNPM1, FDA label safety population (N = 112) [35] and, for the phase II and earlier phase Ib estimates, the published KOMET-001 reports [32,33]; ziftomenib KMT2Ar, phase Ib cohorts [33]; bleximenib, cAMeLot-1 dosed population [38,40]; enzomenib, all treated patients (N = 84) [42]. Grade ≥ 3 cytopenia duration is not uniformly reported in the primary sources and is shown qualitatively where available.

Trial/Agent	Population	N (Enrolled/Evaluable)	Sponsor	RP2D	CR + CRh	ORR	Median DoR	QTc (any/≥3)	DS (any/≥3)	Grade ≥ 3 Cytopenia Duration
AUGMENT-101/Revumenib	KMT2Ar	413 (overall)/104 (efficacy)	Syndax Pharmaceuticals	163 mg Q12H + CYP3A4i; 276 mg Q12H w/o	21.2%	—	6.4 mo	26%/14%	28%/16%	~1–2 mo to recovery among responders
AUGMENT-101 C2C/Revumenib	mNPM1	413 (overall)/64 (efficacy)	Syndax Pharmaceuticals	163 mg Q12H + CYP3A4i; 276 mg Q12H w/o	23.4%	46.9%	4.7 mo	36%/17%	25%/12%	~1–2 mo to recovery among responders
KOMET-001/Ziftomenib	mNPM1	112 (pooled)/92 (Ph II)	Kura Oncology/Kyowa Kirin	600 mg QD	23% (Ph II); 25% (pooled); 21.4% (FDA, N = 112)	33% (Ph II); 35% (pooled)	3.7–5.0 mo	12%/8%	26%/13% (FDA label); 25%/15% (Ph II); earlier Ph Ib ~12–15%/~5%	Not uniformly reported
KOMET-001/Ziftomenib	KMT2Ar	53 (phase 1b)	Kura Oncology/Kyowa Kirin	600 mg QD	—	~17–45% (early cohorts)	Not mature	Not reported	~38%/25–30%	Not mature
cAMeLot-1/Bleximenib	KMT2Ar + mNPM1 (pooled RP2D)	146 dosed/21 (RP2D efficacy)	Janssen Research & Development	100 mg BID (50 mg step-up)	33.3%	47.6%	6 mo	No signal reported	14%/6–9%	Dose-modification driven at 150 mg BID
DSP-5336 Ph I/II/Enzomenib	KMT2Ar	84 (overall)/15 (KMT2Ar, RP2D)	Sumitomo Pharma	300 mg BID	40.0%	73.3%	NR (7.0 mo range)	Rare/1%	10.7%/0%	No DS prophylaxis used
DSP-5336 Ph I/II/Enzomenib	mNPM1	84 (overall)/7 (mNPM1, RP2D)	Sumitomo Pharma	300 mg BID	42.9%	57.1%	Not reported	Rare/1%	10.7%/0%	No DS prophylaxis used

**Table 3:** Summary of combination-regimen trials of menin inhibitors in AML. Abbreviations: AZA, azacitidine; DLT, dose-limiting toxicity; MTD, maximum tolerated dose; ND, newly diagnosed; R/R, relapsed/refractory; VEN, venetoclax. Notes: sample sizes are the enrolled/treated populations reported by each source and response definitions differ (e.g., CR, CR/CRh, composite CR/CRi/MLFS); the data derive from early-phase, non-randomized studies and cannot be compared across trials or with VEN/AZA alone.

Trial	Regimen	Setting/Population	N (Enrolled)	Sponsor	Key Efficacy	DS (any/≥3)	Hematologic Toxicity
Beat AML substudy	Revumenib + VEN/AZA	ND, mNPM1/KMT2Ar, ≥60y unfit for intensive chemo	43	University of North Carolina/Beat AML consortium	ORR 88.4%; CR 67.4%; 100% flow MRD-neg among responders	18.6%/4.7%	Grade ≥ 3 febrile neutropenia 26%; no MTD identified
SAVE	Revumenib + VEN + decitabine/cedazuridine	R/R, mNPM1/KMT2Ar/NUP98r, all-oral	33	MD Anderson Cancer Center	ORR 82%; CR/CRh 48%; MRD-neg 65% of responders	Generally well tolerated	Not separately reported
KOMET-007 (ND cohort)	Ziftomenib + 7 + 3	ND, intensive-chemo-eligible, mNPM1/KMT2Ar	82 (49 NPM1 + 33 KMT2Ar)	Kura Oncology/Kyowa Kirin	96% alive on study (NPM1); CR 85% (KMT2Ar)	Consistent with 7 + 3 background	Febrile neutropenia 60%, expected for 7 + 3 backbone
KOMET-007 (R/R cohort)	Ziftomenib + VEN/AZA	R/R, mNPM1/KMT2Ar, doses 200–600 mg	76 (49 NPM1 + 27 KMT2Ar)	Kura Oncology/Kyowa Kirin	ORR 68% (mNPM1); 6-mo OS 77% (mNPM1), 43% (KMT2Ar)	Manageable	Not separately reported
Bleximenib + VEN/AZA (Ph I)	Bleximenib + VEN/AZA	R/R (n = 85) and ND (n = 40 targeted), KMT2Ar/mNPM1/NUP98-NUP214	125 targeted	Janssen Research & Development	R/R ORR 70–91.7%; ND ORR 75–93.8%; exploratory: no acquired MEN1 mutations detected in 19 evaluable R/R patients (small subgroup)	Not separately reported at 100 mg BID	No DLTs at 100 mg BID

**Table 4:** Ongoing or planned randomized controlled trials of menin inhibitors in AML. No results are yet available from any of these studies. Abbreviations: AZA, azacitidine; ND, newly diagnosed; OS, overall survival; VEN, venetoclax.

Trial (Acronym)	NCT Identifier	Design	Population	Primary Endpoint(s)	Status (as of 2026)
EVOLVE II	NCT06652438	Phase III, randomized, revumenib + AZA/VEN vs. AZA/VEN alone	ND mNPM1 or KMT2Ar AML, ineligible for intensive chemotherapy	Event-free survival/overall survival	Recruiting
cAMeLot-2	NCT06852222	Phase III, randomized, double-blind, placebo-controlled; bleximenib + VEN/AZA vs. placebo + VEN/AZA	ND KMT2Ar or mNPM1 AML, ineligible for intensive chemotherapy	Overall survival	Recruiting
KOMET-007 (randomized portion)	NCT05735184 (expansion)	Phase I/randomized expansion, ziftomenib-based regimens vs. standard backbones under evaluation	ND and R/R mNPM1/KMT2Ar AML	Complete remission rate; safety; OS (exploratory)	Enrolling/regimen selection ongoing

## Data Availability

Data sharing is not applicable. No new data were created or analyzed in this study.
